# Detailed Dissection and Critical Evaluation of the Pfizer/BioNTech and Moderna mRNA Vaccines

**DOI:** 10.3390/vaccines9070734

**Published:** 2021-07-03

**Authors:** Xuhua Xia

**Affiliations:** 1 Department of Biology, University of Ottawa, Ottawa, ON K1N 6N5, Canada; xxia@uottawa.ca; 2 Ottawa Institute of Systems Biology, University of Ottawa, Ottawa, ON K1H 8M5, Canada

**Keywords:** SARS-CoV-2, mRNA vaccine, translation initiation, codon optimization, translation termination, RNA secondary structure, RNA stability

## Abstract

The design of Pfizer/BioNTech and Moderna mRNA vaccines involves many different types of optimizations. Proper optimization of vaccine mRNA can reduce dosage required for each injection leading to more efficient immunization programs. The mRNA components of the vaccine need to have a 5′-UTR to load ribosomes efficiently onto the mRNA for translation initiation, optimized codon usage for efficient translation elongation, and optimal stop codon for efficient translation termination. Both 5′-UTR and the downstream 3′-UTR should be optimized for mRNA stability. The replacement of uridine by N1-methylpseudourinine (Ψ) complicates some of these optimization processes because Ψ is more versatile in wobbling than U. Different optimizations can conflict with each other, and compromises would need to be made. I highlight the similarities and differences between Pfizer/BioNTech and Moderna mRNA vaccines and discuss the advantage and disadvantage of each to facilitate future vaccine improvement. In particular, I point out a few optimizations in the design of the two mRNA vaccines that have not been performed properly.

## 1. Introduction

The two most frequently used SARS-CoV-2 vaccines, from Pfizer/BioNTech [1] and Moderna [2], respectively, are both mRNA vaccines. The sequence of Pfizer/BioNTech’s BNT-162b2 is publicly available [3], and the sequence of Moderna’s mRNA-1273 has recently been sequenced [4]. Both mRNA encodes the same S-2P protein [5,6] which differ from the spike protein in the reference SARS-CoV-2 genome (NC_045512) by two amino acids, i.e., amino acids KV at sites 986 and 987 were replaced by PP to stabilize the resulting spike protein in the prefusion state to train the host immune system to recognize the virus before its entry into the host cell [7,8].

While the Pfizer/BioNTech’s BNT162b2 mRNA and Moderna’s mRNA-1273 share the same amino acid sequence, they differ in many other ways, such as the design of 5′-UTR, codon optimization and 3′-UTR. Translation initiation is typically the limiting step in translation, and its efficiency depends heavily on how rapidly the 5′-UTR can load ribosome onto the mRNA [9]. Translation elongation becomes rate-limiting when translation initiation is highly efficient [10,11]. Optimization of vaccine mRNA for efficient translation can decrease the copies of vaccine mRNA needed to be carried into host cells.

As mammalian host cells attack unmodified exogeneous RNA [12,13], all U nucleotides were replaced by N1-methylpseudouridine (Ψ) [14,15]. However, Ψ wobbles more in base-pairing than U and can pair not only with A and G, but also, to a lesser extent, with C and U [16]. This is likely to increase misreading of a codon by a near-cognate tRNA. When nucleotide U in stop codons was replaced by Ψ, the rate of misreading of a stop codon by a near-cognate tRNAs increased [17]. Such readthrough events would not only decrease the number of immunogenic proteins, but also produce a longer protein of unknown fate with potentially deleterious effects.

I performed a detailed dissection and critical evaluation of different optimization strategies of vaccine mRNA from Pfizer/BioNTech and Moderna, from 5′-UTR to 3′-UTR, and highlight their similarity and differences. By using genomic and transcriptomic data, I pointed out a few inappropriately performed optimizations in the design of the two mRNA vaccines. The objective is to facilitate the development of better strategies in vaccine mRNA optimization in the future.

## 2. Materials and Methods

### 2.1. Sequence Data

Much of mRNA optimization is based on contrast between highly expressed protein-coding genes and average protein-coding genes. Ribosomal protein genes have been used throughout the paper as representatives of functionally important and highly expressed genes. HUGO gene nomenclature committee (HGNC at https://www.genenames.org/, accessed on 20 June 2021) lists 35 small and 54 large ribosomal protein genes. These genes were then downloaded from www.ncbi.nlm.nih.gov/gene (accessed on 20 June 2021). Some of the downloaded genes are pseudogenes, e.g., rpL21 and two rpL7a isoforms. RPS4Y2 is also annotated as a pseudogene in NC_000024. These genes, as well as some other genes that are homologous to ribosomal protein genes but are not expressed in most tissues (RPL10L, RPL39L, and RPL3L), were excluded. Only ubiquitously expressed ribosomal protein genes (33 RPS and 50 RPL genes) were included. The supplemental sequence file in FASTA format (RP_Longest_isoform_ubiquitous.fas) contains the longest splice isoform for each ribosomal protein genes. Some results in the paper include all splice isoforms so that total number of coding sequences (CDSs) is greater than 83.

Human genomes (chromosomes 1 to 22, X and Y (NC_000001-NC_000024) were downloaded from NCBI. The 11,327 annotated CDSs (including splicing isoforms) in chromosome 1 (Chr01) were used as a representative set of human genes to contrast against ribosomal protein genes (as a representative set of highly expressed genes). The nucleotide frequencies of all introns in chromosomes 18 to 22 (0.2640, 0.2178, 0.2262, and 0.2920 for A, C, G and T, respectively) were used as a proxy of background frequencies in computing position weight matrix.

The reference genomes of SARS-CoV and SARS-CoV-2 (NC_004718 and NC_045512, respectively) were downloaded from GenBank, and so were other related coronaviruses isolated from bats (MN996532, MG772933, MG772934). The coronavirus sequence isolated from pangolin (pangolin|EPI_ISL_410721|2019) was downloaded from GISAID. The vaccine mRNA BNT-162b2 is publicly available [3]. The sequence of mRNA-1273 was taken from Jeong et al. [4], and should be considered putative. However, the 5′-UTR of this putative sequence is identical to one of the 5′-UTR sequences (SEQ ID NO 181) in a Moderna patent [18]. Similarly, the 3′-UTR of this sequence is identical to one of the 3′-UTR sequences (SEQ ID NO 21) in another Moderna patent [19], except that the seventh triplet is UAG in the putative sequence instead of AUG. In this context, it may be safe to assume that the putative sequence is the real mRNA-1273.

### 2.2. Tissue-Specific Gene Expression

As the two mRNA vaccines are administered through muscle injection, it is relevant to characterize features of highly expressed muscle genes. The Human Protein Atlas (www.proteinatlas.org, accessed on 20 June 2021) contains tissue-specific gene expression data. The rna_tissue_consensus.tsv.zip file from the site contains gene expression data from 62 tissues. Gene expression of 19,670 human protein-coding genes (including 13 mitochondrial protein-coding genes) were characterized in skeletal muscle. I sorted the genes by their expression and took the top 50 as representative genes highly expressed in the skeletal muscle. I included a supplemental file HEG50_Muscle.fas that contains the coding sequences of the longest isoform of these 50 highly expressed skeletal muscle genes.

**Figure 1:**
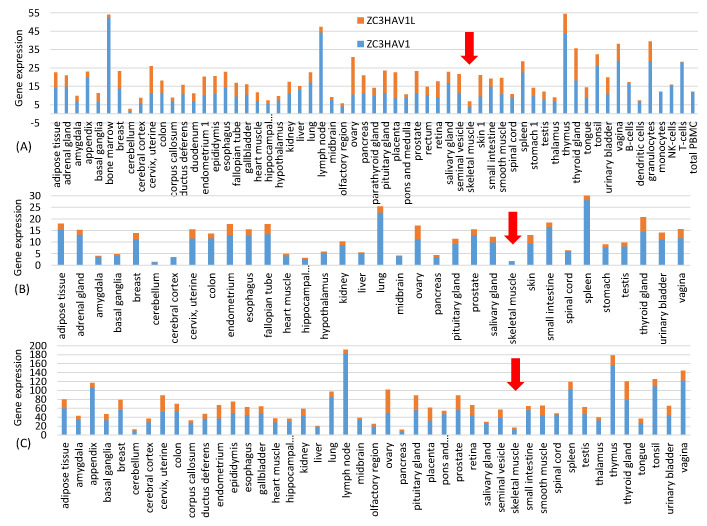
Tissue-specific gene expression of ZAP (*ZC3HAV1* and its long form *ZC3HAV1L*) extracted from three gene expression files from Human Protein Atlas (www.proteinatlas.org, accessed on 20 June 2021) representing three independent transcriptomic experiments: (**A**) proteinatlas.tsv, (**B**) rna_tissue_gtex.tsv and (**C**) rna_tissue.fantom.txv. ZAP expression is low in skeletal muscle (pointed to by the red arrow) in all three data sets. The horizontal axis is alphabetically sorted. The three data sets do not include the same types of tissues.

Data for Figure 1 are from three independent quantifications of tissue-specific gene expression represented by three files in the Human Protein Atlas: proteinatlas.tsv, rna_tissue_gtex.tsv and rna_tissue_fantom.tsv. The tissue-specific expression of the zinc finger antiviral protein ZAP (*NC3HAV1* and its long form *ZC3HAV1L*) were extracted from each of the three files and plotted in Figure 1. The near absence of ZAP in muscle cells suggests that spike mRNAs in the vaccine injected into muscle cells will not be subject to ZAP-mediated RNA degradation.

### 2.3. Sequence Compilation and Analysis

I used DAMBE [20] to extract coding sequences, stop codons, and sequences upstream and downstream of coding sequences. Codon frequencies, codon adaptation index [21,22], index of translation efficiency (I_TE_) [11], position weight matrix (PWM) [23,24], and minimum folding energy (MFE) were also computed from DAMBE. MFE calculation in DAMBE uses functions in the Vienna RNA fold library [25].

### 2.4. Viral Subgenomic mRNA from Transcriptomic Data

Given that mRNA sequences transcribed naturally by SARS-CoV-2 viruses could potentially shed light on vaccine mRNA optimization, I downloaded SARS-CoV-2 transcriptomic data [26] from NCBI’s SRA database. The set of transcriptomic data contains good-quality samples (e.g., GC-26/66 corresponding to SAR file SRR11886744.sra) and poor-quality samples (e.g., GC-55/68 corresponding to SRR11886743.sra). I downloaded SRR11886744.sra and analyzed subgenomic mRNA for the spike protein naturally produced by proliferating SARS-CoV-2. The 5′-UTR from naturally produced spike mRNA was derived from the analysis.

## 3. Results and Discussion

### 3.1. Codon Optimization for Translation Elongation Efficiency

There are two levels of codon optimization. The first involves compound codon families. For example, SARS-2-S in the reference genome (NC_045512) contains 42 Arg residues, of which 30 are encoded by AGR codons and only 12 are encoded by CGN codons (S_Ref_ column in Table 1). This avoidance of CGN codons makes evolutionary sense given that the host zinc finger antiviral proteins (ZAP, gene name *ZC3HAV1*) target CpG dinucleotides in viral RNA and recruit cellular RNA degradation complexes to degrade the viral RNA genome [27,28,29]. However, human genes use CGN more frequently than AGR codons for encoding Arg. Among the ribosomal protein genes (33 RPS and 50 RPL) known to be highly expressed, 64.2% of the Arg residues are encoded by CGN codons. BNT-162b2 and mRNA-1273 reduced AGR codons by 8 and 28, respectively, with the corresponding increase in CGN codons.

One might ask if the resulting increase in CpG dinucleotides would result in rapid degradation of the vaccine mRNA after being delivered into the host cell through the ZAP-mediated RNA degradation pathway [27,28,29]. This is not a concern with the intramuscular injection because, according to three sets of gene expression data from Human Protein Atlas at http://www.proteinatlas.org (accessed on 20 June 2021) [30], ZAP is almost absent in skeletal muscle (Figure 1). This highlights one advantage of mRNA vaccines because it has many different but convenient routes for vaccine administration, including subcutaneous, intramuscular, intradermal, intratracheal, intravenous and intraperitoneal routes [31]. The high CpG in the vaccine mRNA provides two additional benefits. First, GC-rich mRNAs tend to be more stable than AU-rich mRNAs [32]. Second, in the unlikely case when the vaccine mRNAs were recombined into a SARS-CoV-2 virus, the result would not be a virus with an optimized spike protein gene, but a segment of CpG-rich RNA that would be targeted by host ZAP for degradation.

The compound codon family for Leu is optimized similarly. Highly expressed human ribosomal protein genes encode 81% of Leu by CUN codons. For this reason, almost all UUR codons for Leu were recoded to CUN codons in both vaccine mRNAs (Table 1). The compound codon family for Ser introduces a new twist. Both codon subfamilies are used roughly equally for encoding Ser. However, it is easier to optimize the AGY subfamily because AGC is clearly the preferred codon over AGU. Note that mutation bias would have favored U-ending codons because the frequency of U in introns is higher than that of C (0.2920 for U and 0.2178 for C, Table 1), but highly expressed ribosome protein genes prefer AGC over AGU (144 for AGC and 95 for AGU, Table 1). In contrast, the UCN subfamily has both UCC and UCU used frequently. For this reason, many Ser codons UCN were recoded to AGC in the vaccine mRNAs, especially in mRNA-1273 (Table 1).

The second level of codon optimization is within-family optimization. Two strategies have been used. The first, referred to hereafter as the fundamentalist strategy, is simply to replace all codons by the major codon. Which codon is a major codon depends conceptually on two criteria: (1) the codon is preferred by highly expressed genes, and (2) it is decoded by the most abundant tRNA. However, superficial application of these two criteria can lead to mistakes. I will take the CGN codon family for Arg to show an incorrect optimization of the two mRNA vaccines.

The designers of both vaccines considered CGG as the optimal codon in the CGN codon family and recoded almost all CGN codons to CGG. This choice of CGG as the optimal codon seemingly resulted from application of both criteria above. First, the EMBOSS [33] compilation of codon usage, which is frequently used in codon optimization, shows that CGG is used slightly more frequently than CGC. Second, CGG seems to have more tRNA decoding it than other synonymous CGN codons. A human genome contains seven tRNA^Arg/ACG^ genes (where superscripted ACG is the anticodon, with A deaminated to inosine I) to decode CGY codons, four tRNA^Arg/CCG^ genes to decode CGG codons and six tRNA^Arg/UCG^ genes to decode CGA and CGG (through wobble pairing at third codon site). Assuming that tRNA abundance is well correlated with tRNA gene copy number, which is true for *Saccharomyces cerevisiae* [34] but not known for other eukaryotes, one can infer that CGG is translated by more tRNAs genes (four tRNA^Arg/CCG^ genes six tRNA^Arg/UCG^ genes) than other codons and therefore is the major codon based on the two criteria. The two vaccines recoded nearly all CGN codons to CGG (Table 1).

The reasoning above involving tRNA gene copy number is problematic. Nearly half of human tRNA genes are not expressed [35], so we cannot use tRNA gene copy number as a proxy of tRNA abundance in the cellular tRNA pool. For this reason, codon preference by highly expressed genes relative to lowly expressed genes is a better operational criterion for codon optimization. The codon compilation of human genes in EMBOSS [33] was done in 1993 and 1994 and did not aim to include only the highly expressed, so the slightly higher usage of CGG than CGC may simply be due to mutation bias (The frequency for nucleotide G is consistently higher than that of C in human introns).

There are two lines of evidence suggesting that CGG is not the optimal codon. The first involves the codon usage of human ribosomal protein genes (“RP” in Table 1) which are known to be highly expressed. These genes prefer CGC codons (Table 1). The second and more direct evidence is from codon usage of genes highly expressed in skeletal muscle cells (which are relevant here because the vaccine mRNA is injected and carried by the lipid nanoparticles into skeletal muscle cells to be translated, although vaccine mRNA could also be carried to some other tissues). I chose 50 genes most highly expressed in skeletal muscles from the consensus expression data set in Human Protein Atlas at http://www.proteinatlas.org (accessed on 20 June 2021) [30], but excluded those with CDSs with fewer than 300 codons. The remaining 26 genes (Table 2), including the most muscle-specific genes such as titin (*TTN*), actin (*ACTA1*) and myosin (*MYH1*), use CGC codons significantly more than CGG codons (Paired sample *t*-test, *t* = 3.075, DF = 25, *p* = 0.0034, 2-tailed test). Therefore, the CGC codon preferred by ribosomal protein genes are also preferred by highly expressed muscle genes. Other protein-coding genes that are highly expressed are the two isoforms of human elongation factor 1α (*hEF1A1* and *hEF1A2*), and poly(A)-binding protein (*hPABPC1*). They also use more CGC than CGG (CGC:CGG are 3:0 for *hEF1A1*, 8:6 for *hEF1A2*, and 14:4 for *hPABPC1*). These multiple lines of evidence suggest that CGC is a better codon than CGG. The designers of the mRNA vaccines (especially mRNA-1273, Table 1) chose a wrong codon as the optimal codon.

Optimization of other codon families are straightforward. For 2-fold R-ending codons, background mutation bias, as reflected by nucleotide frequencies of introns in human genome, favors A-ending codons, but ribosomal protein genes consistently favor G-ending codons in every 2-fold R-ending codon family. Consequently, G-ending codons were taken as the optimal codon in the two mRNA vaccines (Table 3 for GAR codons encoding Glu). For 2-fold Y-ending codons, the background mutation favors U-ending codons, but ribosomal protein genes favor C-ending codons, so C-ending codon is the optimal codon. There is another reason for recoding U-ending codons to C-ending codons. All U nucleotides in the two mRNA vaccines were replaced by N1-methylpseudouridines (Ψ) which can wobble with all for nucleotides and, therefore, should not be used in 2-fold codon families. For example, GAΨ encoding Asp could pair with the anticodon of tRNA^Glu^ leading to nonsynonymous substitutions. C-ending codons do not have this problem, which serves as another reason for recoding U-ending codons to C-ending codons.

The second strategy in codon optimization, referred to hereafter as the liberal strategy, is simply a less extreme version of the fundamentalist strategy that replaces all synonymous codons by the optimal codon. Suppose a synonymous codon family NNR with NNG decoded by tRNA-1 and NNA decoded by tRNA-2. Additionally, suppose that tRNA-1 is twice as abundant as tRNA-2 and that highly expressed genes favor NNG codon over NNA codon. The fundamentalist strategy is to replace all codons by NNG. The liberal strategy is based on the following rationale. When a cell is full of mRNA with NNG codons, tRNA-1 will be under such a high demand that it may become less available than tRNA-2, although there are twice as many tRNA-1 in the cell than tRNA-2. For this reason, it might be more optimal to keep some codons decoded by tRNA-2.

These two strategies are exemplified by the codon optimization involving GAR codons encoding Glu (Table 3). The SARS-CoV-2 reference genome (NC_045512) has 34 GAA codons and 14 GAG codons in its spike protein gene. Moderna’s mRNA-1273 has taken the fundamentalist strategy and replaced all GAA codons by GAG. In contrast, Pfizer/BioNTech’s BNT-162b2 took the liberal strategy, and left 14 GAA codons unchanged (Table 3). Moderna has consistently applied the fundamentalist strategy for all codon families in mRNA-1273, whereas Pfizer/BioNTech has consistently used the liberal strategy in codon optimization for BNT-162b2. There is no systematic evaluation of these two codon optimization strategies in translation efficiency. Given the difference in dosage (100 μg with mRNA-1273 and 30 μg with BNT-162b2) and the equivalence in efficacy, one may assume that an injection of Pfizer/BioNTech or Moderna vaccine produces the same number of the encoded spike proteins. This would imply that mRNA in the Pfizer/BioNTech vaccine on average likely produces about 3.3 times as many proteins as an mRNA in the Moderna vaccine.

The codon optimization applied to BNT-162b2 and mRNA-1273 leads to a much increased codon adaptation index (CAI) [21,22] and index of translation efficiency (I_TE_) [11,36] for the two vaccine mRNAs. The S gene from natural coronaviruses have CAI < 0.7 for their spike protein CDS, but the two codon-optimized spike CDSs have CAI equal to 0.94925 and 0.97939, respectively (Table 4). I_TE_ is a generalized CAI taking into consideration of background mutation bias [11]. Its values are similarly much higher in the two vaccine mRNAs than in natural viruses. The maximum CAI and I_TE_ values are 1.

The smaller value of CAI and I_TE_ values for BNT-162b2 than mRNA-1273 might give an impression that BNT-162b2 is less codon-optimized than mRNA-1273. This is not necessarily true. As I mentioned before, mRNA-1273 was codon-optimized with the fundamentalist strategy (i.e., replacing all or almost all synonymous codons by the optimal codon), whereas BNT-162b2 was optimized with the liberal strategy which is less extreme than the first. The fundamentalist strategy will necessarily generate higher CAI or I_TE_ values than the liberal strategy. However, the liberal strategy might lead to more efficient translation elongation if there are too many codons demanding the most abundant tRNA, as I discussed before.

### 3.2. Codon Optimization for Translation Accuracy

The codon optimization in the previous section suffers from the lack of consideration for translation accuracy [36,37]. Take Asn codons AAC and AAU in *E. coli* {*XE* “E. coli”} for example. AAC is a major codon (heavily used by highly expressed genes and decoded by the most abundant isoacceptor tRNA {XE “tRNA: isoacceptor”}{XE “isoacceptor tRNA”}) whereas AAU is a rarely used minor codon. Highly expressed *E. coli* genes use AAC almost exclusively to encode Asn, so one could argue that the overuse of AAC is driven by selection for translation efficiency. However, AAC and AAU also differ in misreading rate, in particular by tRNA^Lys^, which ideally should decode only AAA and AAG codons but does misread AAC and AAU, leading to Asn replaced by Lys. This misreading error rate is six times greater for AAU than for AAC, with the error ratio consistently maintained in different experimental settings, e.g., under both Asn-starved and non-starved conditions [38], or with Streptomycin used to inhibit translation [39]. Therefore, the overuse of AAC by highly expressed *E. coli* genes could be driven either by selection for increased translation efficiency or increased translation accuracy or both.

Akashi [37] attempted to disentangle the effect of selection on translation efficiency and accuracy. He classified amino acid sites into conserved sites (assumed to be functionally important) and variable sites (assumed to be of limited importance). If codon adaptation is due to selection for translation efficiency, then all codons in the gene should be subject to similar selection regardless of whether the codon is in a functionally important or unimportant site. In contrast, if codon adaptation is driven by selection for translation accuracy, then the selection is stronger in functionally important sites than in functionally unimportant sites. This implies greater select effect on functionally important codon sites than functionally unimportant codon sites. He found greater codon adaptation in conserved amino acid sites than in variable amino acid sites. This is consistent with his inference that the difference between the conserved and variable sites has resulted from selection for accuracy.

The observation, however, is also consistent with selection for translation efficiency. Take lysine codons (AAA and AAG) and glutamate codons (GAA and GAG) for example. Suppose that AAA codon can be decoded more efficiently than AAG, and GAG decoded more efficiently than GAA. Additionally, suppose that a highly expressed ancestral gene has evolved strong codon adaptation with lysine coded mainly by AAA and glutamate coded mainly by GAG. Now, some lysine sites might happen to experience nonsynonymous substitution {XE “nonsynonymous substitution”}s from AAA to GAA. These sites are now designated as variable (functionally unimportant) sites and are occupied by a minor codon GAA. This would result in an association between “poor codon adaptation” and variable (functionally unimportant) sites that has little to do with translation accuracy. Akashi [37] discussed this problem but did not provide a definitive solution.

There are two approaches to optimize codon usage for accuracy. The first is to empirically characterize the decoding error rate for each synonymous codon in skeletal muscle cells, and to choose the codon with the lowest error rate. For mRNA to be translated in *E. coli,* then recoding AAU to AAC would increase accuracy because AAC has a misreading error six times smaller than AAU. An alternative is again to follow the codon usage of functionally important and highly expressed genes, such as ribosomal proteins or highly expressed genes in skeletal muscle cells in Table 2. It is important for vaccine mRNA to be translated accurately because misincorporation of the wrong amino acids would confuse our immune system in target recognition.

### 3.3. Translation Initiation Signal

Optimum codon usage without efficient translation initiation does not increase protein production [10,11] because translation initiation is often the rate-limiting step. Efficient translation initiation in mammalian species depends mainly on two factors [40,41]: (1) the Kozak consensus [42,43,44], and (2) the secondary structure that may embed the Kozak consensus to obscure these essential translation initiation signals [9,41,45]. These factors contribute to the efficiency of ribosomes being properly positioned at start codon to transit from translation initiation to elongation. I disregard the nature of start codon as a relevant factor contributing to translation initiation efficiency because there is little variation in start codon usage in mammalian genes. For example, among 11,327 annotated protein-coding genes and their splice isoforms in human chromosome 1 (Chr01, NC_000001.11), only five genes (OAZ3, FNDC5, FNDC5, RNF187 and WDR26) have one of its isoforms featuring a non-AUG start codon.

#### 3.3.1. Human Translation Initiation Consensus

The Kozak consensus for mammalian genes that enhances translation initiation is GCCRCC**AUG**G [40,46], where **AUG** is the start codon. To corroborate this consensus, I show in Table 5 the site-specific nucleotide frequencies flanking the start codon AUG for all protein-coding genes (including isoforms) from human Chr01. The associated position weight matrix [23,24,47,48], using intron nucleotide frequencies (0.26398, 0.21777, 0.22622 and 0.29203 for A, C, G, and T, respectively) as background frequencies, shows a site-specific pattern consistent with the GCCRCC**AUG**G consensus. This pattern is consistent for genes from other human chromosomes, and stronger in highly expressed genes than lowly expressed genes.

The two mRNA vaccines both used GCCACC**AUG**, but not the codon after the start codon AUG, for two good reasons. First, while the -3R (site 4 in the first column of Table 5) has been demonstrated repeatedly to enhance translation initiation, the effect of +4G (site 10 in Table 5), as well as nucleotides downstream, on translation initiation has been inconclusive [46,49,50,51]. The preponderance of +4G was explained by the amino acid constraint hypothesis [52,53] as follows. About 60% of the proteins experience N-terminal methionine excision (NME) which requires a small and nonpolar amino acid such as alanine and glycine. Alanine is encoded by GCN and glycine by GGN, leading to a high frequency of G at the +4 site. There is little evidence that +4G and downstream nucleotides contribute to translation initiation. Second, the second amino acid in the spike protein is phenylalanine, which ensures that NME does not happen. Changing it to GCG (encoding alanine) would result in NME leading to unpredictable changes in the S protein. For these reasons, the first codon is not considered in Kozak consensus optimization.

#### 3.3.2. 5′-UTR and Secondary Structure Flanking the Start Codon

5′-UTR serves two key functions: to stabilize mRNA and to facilitate scanning by small ribosome subunit to localize the start codon. There are three strategies in optimizing 5′-UTR in mRNA vaccine development. The first is simply to take the 5′-UTR of a highly expressed human gene, such as the 5′-UTR of human α-globin genes. The second is to use the native mRNA for SARS-2-S. These first two strategies assume that the optimization done by natural selection can be extrapolated to translation in muscle cells. The third is by systematic evolution of ligands by exponential enrichment (SELEX) that has been used in optimizing the 3′-UTR [54] but could be adapted for optimizing 5′-UTR as well. For designing a vaccine against a pandemic, rapid development is the most important, so the first two approaches seem most reasonable.

**Figure 2:**
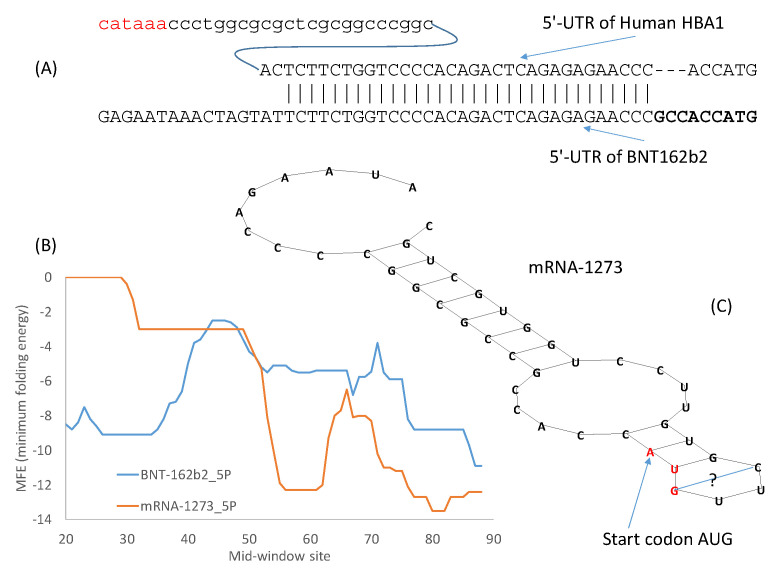
5′-UTR and secondary structure of the two vaccine mRNAs. (**A**) 5′-UTR of BNT162b2 was partially taken from human α-globin gene where the 37-nt 5′-UTR is shared by *HBA1* and *HBA2 mRNAs.* The upstream TATA box is colored in red, and the Kozak consensus highlighted in bold. (**B**) Secondary structure stability measured by MFE (minimum folding energy) over a sliding windows of 40 nt, with start codon AUG at Mid-window sites 55–57. A strong secondary structure (small MFE) is visible in sequences flanking the start codon in mRNA-1273. (**C**) Visualization of the secondary structure embedding the start codon in mRNA-1273.

The design of the 5′-UTR of BNT162b2 took the first approach by incorporating the 5′-UTR of human α-globin (5′-UTR is identical between human HBA1 and HBA2) with a minor modification of the Kozak consensus (Figure 2A). As shown before in Table 5, the optimal Kozak consensus is GCCACCAUG which is used to replace the original ACCAUG (Figure 2A). This follows naturally from earlier approaches of designing the 3′-UTR by incorporating regulatory elements for stability from human α-globin and β-globin [13]. One additional advantage of using the 5′-UTR of a highly expressed human gene is that such 5′-UTRs are almost invariably devoid of upstream AUG that could interfere with translation initiation.

As demonstrated in previous studies on translation initiation in yeast [55] and in mouse cell lines [56], stable secondary structure in the 5′-UTR before or flanking the start codon decreases protein production. However, secondary structure downstream of the start codon tend to enhance recognition of start codons by eukaryotic ribosomes [57]. BNT162b2 has little secondary structure flanking the start codon that is located at mid-window sites 55–57 (Figure 2B). However, Moderna’s mRNA-1273 is peculiar in having a secondary structure flanking the start codon (Figure 2B) that is visualized in Figure 2C. The MFE for the 40 nucleotides with the start codon in the middle is -12.3 for mRNA-1273 at 37 °C.

The long stem in Figure 2 has a G/U base pair to close the stem. G/U base pairs are usually too weak to close a stem. However, all U nucleotides in the mRNA vaccines have been modified to N1-methyl-pseudouridine (Ψ) [14] to reduce immune reaction towards mRNA and to increase protein production [2,13,58]. G/Ψ base pairs are expected to be stronger than G/U base pairs [16].

Moderna has developed many alternative 5′-UTRs and 3′-UTR sequences listed in two patents [18,19]. The 5′-UTR in Moderna’s mRNA-1273 is V1-UTR (SEQ ID NO 181) [18]. It is made of two elements. The first element is SEQ ID NO 1 in a list of 16,120 sequences in one patent [19]. This element is followed by a GC-rich second element CCCCGGCGCC [18], just before the Kozak consensus ACCAUG. This GC-rich element, and the secondary structure it contributes to (Figure 2C) may increase mRNA stability and translation accuracy by reducing leaky scanning [18].

It is not known if such a secondary structure in the 5′-UTR of mRNA-1273 (Figure 2C) would hamper the cap-dependent scanning for the start codon and result in less efficient translation. However, the 5′-UTR of BNT162b2 derived from 5′-UTR of the human α-globin gene (Figure 2) does appear to be superior over mRNA-1273 in this aspect. As I mentioned before, mRNA-1273 also has a problem in codon optimization (e.g., excessive use of CGG that is not an optimal codon). These factors might jointly impact negatively the translation efficiency of mRNA-1273 and contribute to the requirement of a high dosage of Moderna vaccine (100 μg/dose) relative to that of Pfizer/BioNTech vaccine (30 μg/dose). When an mRNA is not translated efficiently, more of it is needed to produce the same amount of the encoded spike protein.

**Figure 3:**
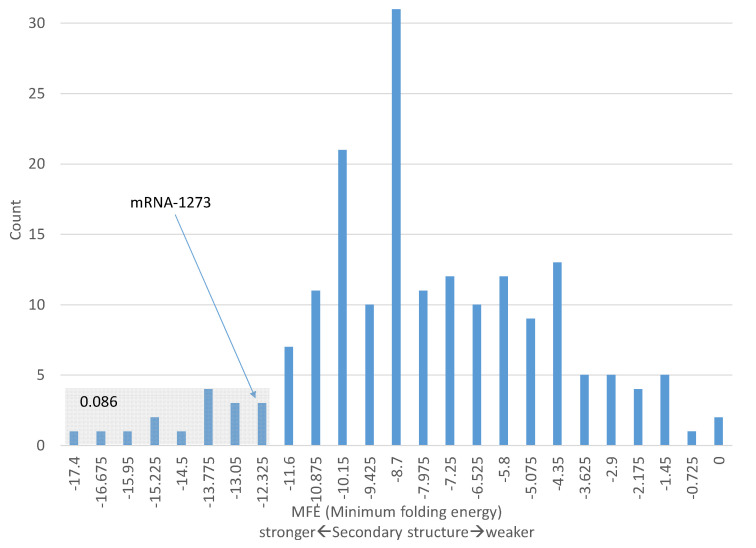
MFE (minimum fold energy) distribution of sequences flanking the start codon AUG in ribosomal protein genes. MFE is calculated from 40-nt with 20 nt upstream of AUG and 20 nt downstream including AUG), based on 186 mRNA variants of 34 RPS and 54 RPL genes. A total of 8.6% of MFE values are equal to, or smaller (more stable secondary structure) than, MFE for mRNA-1273.

One should be cautious in making the interpretation above concerning the secondary structure in Figure 2C). Some genes known to be highly expressed have comparable MFE values in the 40 nucleotides with the start codon in the middle. For example, 8.6% of ribosomal protein genes (including different isoforms) have MFE values for the 40 nt windowd equal or smaller than −12.3 (Figure 3). In other words, the secondary structure of these 8.6% of ribosomal protein genes are equally stable or even more stable than that of mRNA-1723. Therefore, the secondary structure in mRNA-1273 (Figure 2) does not necessarily imply low translation efficiency.

A number of highly expressed human genes have comparable secondary structure at 5′-UTR. For example, the 40-nt window (with 20 nt before the start codon AUG and 20 nt in the 5′ end of CDS) for human α-globin gene has an MFE of −11.1, indicating only a slightly weaker secondary structure relative to the corresponding region in mRNA-1273 with an MFE value of −12.3. However, mammalian highly expressed genes are typically far more GC-rich in sequences flanking the start codon than lowly expressed genes, so the former tend to have more stable secondary structure than the latter. This difference in GC-richness between highly expressed and lowly expressed genes is true both in 5′-UTR immediately upstream of the coding sequence and in 3′-UTR immediately downstream of the coding sequence.

**Figure 4:**
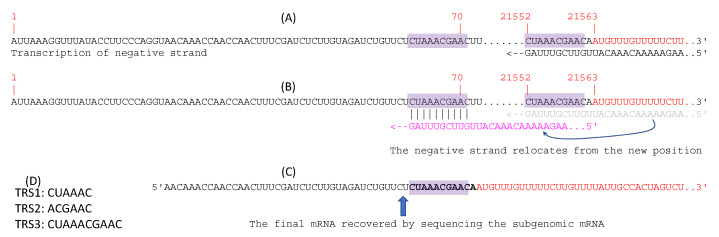
Discontinuous transcription of subgenomic SARS-2-S mRNA. (**A**) SARS-CoV-2 genome (NC_045512) with the leader sequence at sites 1–70, and two shaded transcription regulatory sequence (TRS), with TRS-L at the end of the leader sequence and TRS-B upstream of the coding sequence for the spike protein colored in red. The transcription of the negative strand progresses up to TRS-B and pauses. (**B**) The negative strand shifts location from base-pairing with TRS-B to base-pairing with TRS-L. Transcription of the negative strand continues. (**C**) SARS-2-S mRNA transcribed from the negative subgenomic strand. (**D**) Three frequently used TRS in coronavirus, with the first two likely being degenerate forms of the third.

The 5′-UTR of SARS-2-S mRNA produced natively by SARS-CoV-2 might also be considered as an option. Coronaviruses generate subgenomic mRNAs for translating structural proteins including the spike protein [59,60]. The hypothesized discontinuous transcription is illustrated for the generation of SARS-2-S mRNA (Figure 4) as it has not been done so explicitly before. All subgenomic mRNA transcripts share the same 5′ end leader sequence located at the 5′ end of the genomic sequence (sites 1–70, Figure 4A). This leader sequence includes a transcription regulatory sequence (TRS-L) at its 3′ end (Figure 4A). The coding sequence (CDS) of the spike protein, as well as all downstream coding sequences, features a TRS (TRS-B) upstream of the CDS. The transcription of the subgenomic negative strand pauses at TRS-B, resulting in the negative strand shifting position from base-pairing with TRS-B to base-pairing with TRS-L (Figure 4B). Transcription of the negative strand then resumes. Subgenomic mRNAs for the spike protein gene and all other ORFs downstream share the same leader sequence and the 3′-UTR. This discontinuous mechanism of transcription [26,61] is confirmed by sequencing the subgenomic RNAs from cultured viruses (Figure 4C). There are three frequently used TRS in coronaviruses (Figure 4D). The subgenomic SARS-2-S mRNA, recovered from sequencing, does not include the first 25 nt in the leader sequence.

SARS-CoV-2 uses mainly TRS2 and TRS3. Orf7b and orf10 feature an TRS1 upstream of their CDS but have hardly any detectable subgenomic transcripts. In contrast, other ORFs use TRS2 and TRS3 and have numerous subgenomic transcripts in transcriptomic sequences [26,61]. I found 46 transcriptomic reads matching almost the entire length of the sequence in Figure 4C in the transcriptomic file SRR11886744.sra downloaded from NCBI. This transcriptomic file corresponds to the good-quality sample of GC-26/66 with long reads [26]. This information might be useful in developing a therapeutic agent targeting TRS3 to disrupt this discontinuous transcription.

Note that the native SARS-2-S does not have an optimal Kozak consensus of GCCACCAUG, although it does have -3A which was deemed particularly important for translation initiation [40]. This is typical of genes in mammalian viruses. As argued by Nakamoto [62], when translation initiation sequence does not have a good Kozak consensus in mammals (or Shine-Dalgarno sequence in prokaryotes), a start codon not obstructed by secondary structure becomes crucial for efficient translation initiation. The sequence in Figure 4C does not form secondary structure embedding the start codon.

### 3.4. Translation Termination Signal

In contrast to most prokaryotic species with two release factors (RF1 decoding UAA and UAG and RF2 decoding UAA and UGA), eukaryotic release factor eRF1 recognizes all three stop codons [63,64]. However, this fact does not mean that the three stop codons are equally optimal in eukaryotes. In fact, multiple lines of evidence suggest much difference in termination efficiency and accuracy among the three stop codons. Pfizer/BioNTech’s BNT162b2 mRNA features two consecutive UGA stop codons. Moderna’s mRNA-1273 uses all three different stop codons UGAUAAUAG. Are these the optimal arrangement?

#### 3.4.1. Efficiency and Accuracy in Translation Termination

Termination efficiency is measured by the number of stop codons decoded per unit time, and termination accuracy is measured by proportion of stop codons correctly decoded in contrast to misreading by near-cognate tRNAs (nc-tRNAs). The efficiency is often operationally measured experimentally by the release of a tRNA-linked model peptide on a ribosome complex in the presence of eRF [63,64,65]. These studies suggest that UAA (especially UAAA) is more efficient than other stop signals.

The accuracy is operationally measured by the frequency of misreading of stop codons by nc-tRNAs (leaky termination). The rate of misreading stop codons by tRNAs has been measured for several bacterial species. UGA is the leakiest of the three, with a readthrough frequency of at least 10^−2^ to 10^−3^ in *Salmonella typhimurium* [66] and *E. coli* {*XE* “E. coli”} [67,68]. Readthrough also occurs with stop codons UAG and UAA in bacteria [69,70], but at a lower rate. Natural UAG readthrough frequency is mostly within the range of 1.1 × 10^−4^ to 7 × 10^−3^, depending on the nature of the downstream nucleotides [70,71,72,73]. The readthrough of UAA occurs at frequencies from 9 × 10^−4^ to less than 1 × 10^−5^ [70]. Highly expressed genes in diverse bacterial species strongly prefer UAA codons even in strongly GC-rich genomes with an overwhelming majority of stop codons being UGA [74,75].

Different lines of empirical evidence suggest UAA as the best stop codon in eukaryotes. First, the binding affinity to eRF1 is in the order of UAA > UGA and UAG [63,64], suggesting that UAA is a more efficient stop codon than the other two. Second, highly expressed genes in the yeast prefer UAA stop codons [76]. Empirical data in Table 6 shows that highly expressed mammalian genes also prefer UAA stop codons. This is illustrated by contrasting all coding sequences (CDSs) on human chromosome 1 (Chr01) and human ribosomal protein genes (RP) that are known to be highly expressed. The total of 11,327 CDSs on Chr01 is divided into two groups based on GC content (P_CG_: proportion of C and G in each CDS). CDSs in the low-CG group has P_CG_ between 0.3 and 0.52 and CDS in the high-CG group has P_CG_ between 0.52 and 0.8 (Table 6). UAA is used more frequently in the low-CG group than the high GC group, which is expected from mutation bias. However, Chr01 and RP differ dramatically in UAA usage. Both low-CG and high-CG group of genes in Chr01 feature UGA as the most frequent stop codon. In contrast, RP genes exhibit a strong preference for UAA stop codon (Table 6). In this context, the termination signal UGAUGA in Pfizer/BioNTech’s BNT162b2 and UGAUAAUAG in Moderna’s mRNA-1273 may not be the optimal choice.

One might think that the consecutive stop codons in the two vaccine mRNAs would offer a fail-safe mechanism, given the readthrough observed in the translation of not only yeast genes but also mammalian genes [77,78,79]. For example, human MDH1 has a UGA stop codon that is often translated as Trp (encoded by UGG) or Arg (encoded by CGA and other synonymous codons) leading to an extended protein without frameshifting [77]. An additional in-frame stop codon is expected to prevent the production of such unintended proteins with potentially deleterious effects [80]. However, in many cases, misreading UGA stop codons in prokaryotes is associated with a +1 frameshift [81,82,83,84]. Such frameshifting also occurs in eukaryotes, such as the mammalian *AZ1* gene with a stop codon context UGAU [85], where UGA is the stop codon followed by a U at the +4 site. The first U in the stop codon in translating *AZ1* mRNA is often skipped when the concentration of polyamines is high, resulting in the ribosome reading GAU as the next codon [85]. With such a +1 frameshifting, a downstream in-frame stop codon cannot serve as a fail-safe mechanism. UGA is a poor choice of a stop codon, and UGAU in Pfizer/BioNTech and Moderna mRNA vaccines could be even worse.

#### 3.4.2. Tetranucleotide Termination Signal

It has long been recognized that the translation termination signal is more than a stop codon [86,87], leading to an early proposal of tetranucleotide stop signal including the downstream +4 site [63,65,87,88]. The previous section has already alluded to the association of +4U and +1 frameshift. Recent structural studies of mammalian eRF1 [89,90,91] offered a mechanistic explanation for this tetranucleotide stop signal. The lysine (K) in an NIKS motif in eRF1 interacts with nucleotide U at the first site of a stop codon to induce a conformational distortion so that, instead of the three nucleotides of a stop codon occupying the A site, four nucleotides (stop codon plus the +4 site) are squeezed into the A site, with glycine G626 of eRF1 in close physical proximity to the +4 site.

Nonpolar amino acids typically do not form hydrogen bonds with bases in nucleic acids. However, glycine is an exception. Based on the observed contact between amino acids in a protein and bases in DNA, glycine interacts far more favorably with purine than with pyrimidine [92]. If one may extrapolate this observation from DNA to RNA, then one would predict that G626 would favor purine at the +4 site, i.e., a purine at +4 site is more likely to enhance the stop signal by interacting with G626 in eRF1 than a pyrimidine. Similar predictions can also be made with respect to +5 or +6 sites which, however, have their effect far less consistent than the +4 site on translation termination [65].

It is important to test the prediction concerning the +4 site because, if a purine at the +4 site indeed favors translation termination, then the two mRNA vaccines, with a +4U (actually a +4Ψ), do not have a good termination signal. We can again test the prediction by contrasting nucleotide usage at +4 site between human Chr01 genes RP genes. Functionally important and highly expressed genes such as RP genes are expected to evolve strong termination signals. If a purine at the +4 site is beneficial, then RP genes should on average be more likely to have +4R than Chr01 genes. There are 11,327 annotated Chr01 CDSs (including isoforms), and consequently 11,327 stop codons. These includes 3306 UAA, 2578 UAG and 5443 stop codons (Table 7). The percentage of +4A, +4C, +4G, and +4U for each of the three stop codons are shown in CDSs terminating with UAA, UAG and UGA (Table 7). These percentage values make sense when contrasted with those for the 760 annotated CDSs (including isoforms) of highly expressed RP genes. The hypothesis that highly expressed genes favor +4R is strongly supported, which is consistent across all three stop codons (Table 7). The functionally important and highly expressed RP genes use +4R much more than an average gene represented by Chr01 genes. The difference, when tested by a likelihood ratio test, is highly significant (likelihood ratio chi-square = 931.0514, DF = 17, *p* < 0.000001).

The strong preference of +4R in highly expressed RP genes (Table 7) is consistent with other lines of empirical evidence. Stop codon UGA with a +4C is most prone to readthrough in mammalian genes, especially in the context of UGA CUA [77,78]. Both UGAC and UGAU lead to frequent selenocysteine incorporation [65], suggesting poor decoding of UGA as a stop codon with +4Y. In mammalian genes, the effect of the +4 site is consistent among all three different stop codons in experimental studies, with termination efficiency of UAAR >> UAAY, UAGR >> UAGY and UGAR >> UGAY both in vitro and in vivo [65]. For example, the termination efficiency of UGAC is <20% of UAAA [65]. Table 7 suggests that these early experimental results obtained with specific sequence constructs and translation systems are general and real. In short, the optimal stop signal should be UAAA instead of UGAU/UAGU/UAAU in the two mRNA vaccines.

One caveat in the reasoning above involves the replacement of U by N1-methylpseudouridine (Ψ) in the two vaccine mRNAs. To alleviate host cells to attack exogeneous vaccine mRNA as foreign RNA [13,58], all uridines in the mRNA vaccines were replaced by Ψ [2,14]. Therefore, the stop signals are ΨGAΨGA instead of UGAUGA in Pfizer/BioNTech’s vaccine, and ΨGAΨAAΨAG instead of UGAUAAUAG in Moderna’s vaccine. As Ψ is more promiscuous in base-pairing than U and can pair with both A and G and, to a less extent, with C and U [16], stop codons become more prone to misreading by tRNAs [17]. It is for this reason that both mRNA vaccines use consecutive stop codons as a fail-safe mechanism, with the hope that no frameshifting occurs when the first stop codon fails. However, UGAU is known to cause a +1 frameshifting. It is reasonable to infer that ΨGAΨ may be the same. I have mentioned before that mammalian *AZ1* gene with a stop codon context UGAU is prone to polyamine-induced +1 frameshifting [85]. Such a +1 frameshifting defeats the purpose of having multiple stop codons as a fail-safe mechanism.

### 3.5. The 3′-UTR of mRNA Vaccines

I have previously mentioned different approaches for optimizing 5′-UTR and 3′-UTR. Given sufficient time, the systematic evolution of ligands by exponential enrichment (SELEX) [54] should be the preferred method. However, in an emergency, the alternative approach of borrowing from nature could be more efficient. The 5′-UTR of the Pfizer/BioNTech vaccine mRNA incorporates the 5′-UTR of a human α-globin gene (Figure 2A), which makes sense because α-globin mRNAs are translated very efficiently. The same approach of borrowing from nature has been used for designing 3′-UTR of therapeutic mRNAs, e.g., by incorporating stability regulatory elements from human α-globin and β-globin genes [13]. These stability regulatory elements often form RNA-protein complexes to stabilize mRNA [93,94,95,96,97]. The 5′-UTR and 3′-UTR of globin genes, when ligated to other mRNAs, can confer stability to these mRNAs [54,98,99]. Moderna’s mRNA-1273 “pasted” the 110-nt 3′-UTR of human α-globin gene (*HBA1*) between the last stop codon and a poly(A) tail.The design of the 3′-UTR of the Pfizer/BioNTech mRNA vaccine is a combination of SELEX and borrowing from nature. The objective is to find naturally occurring RNA segments that perform better than the 3′-UTR of human β-globin mRNA [54]. Two RNA segments outperform other alternatives through the SELEX optimization protocol [54]. One of them is from the human mitochondrial 12S rRNA (*mtRNR1*), and the other segment is from human *AES/TLE5* gene. As these two RNA segments were found to have the lowest number of predicted binding sites for miRNAs and the highest hybridization energies [54], two C→U mutations were introduced in the *AES* segment to further increase the binding energy (from MFE = −37 to −39.3 at 37 °C, my calculation from DAMBE). For Pfizer/BioNTech’s mRNA vaccine, the *AES* segment of 136 nt with the two C→Ψ mutations was pasted right after two trinucleotides following the second stop codon. The *mtRNR1* segment of 139 nt was pasted immediately after. This heuristic and empirical approach of borrowing from nature is perhaps more efficient than alternatives in an emergency.

## 4. Conclusions

The two widely used mRNA vaccines, one from Pfizer/BioNTech and the other from Moderna, have been optimized by borrowing from highly expressed human genes. However, there are several inappropriate optimizations. I highlighted and illustrated such cases in the hope that the conceptual framework would facilitate the design of not only vaccines, but also other therapeutic mRNAs.

## Figures and Tables

**Table 1:** Optimization of compound codon families in the two mRNA vaccines.

AA	Codon	RP ^(1)^	Bkground ^(2)^	S_Ref_^(3)^	S_BNT-162b2_^(3)^	S_mRNA-1273_^(3)^
R	AGA	257	0.2640	20	21	0
R	AGG	230	0.2262	10	1	2
R	CGA	169	0.2640	0	0	0
R	CGC	306	0.2178	1	1	0
R	CGG	229	0.2262	2	19	39
R	CGU	171	0.2920	9	0	1
L	CUA	69	0.2640	9	0	1
L	CUC	215	0.2178	12	3	2
L	CUG	440	0.2262	3	105	103
L	CUU	203	0.2920	36	0	1
L	UUA	50	0.2640	28	0	1
L	UUG	172	0.2262	20	0	0
S	AGC	144	0.2178	5	64	96
S	AGU	95	0.2920	17	0	0
S	UCA	80	0.2640	26	0	2
S	UCC	194	0.2178	12	22	1
S	UCG	37	0.2262	2	0	0
S	UCU	180	0.2920	37	13	0

^(1)^ Coding sequences of ribosomal proteins (34 and 53 in the small and large subunits, respectively. Only longest isoform for each gene is included); ^(2)^ Nucleotide frequencies from all introns in human chromosomes 18–22 (NC_000018–NC_000022) as a proxy of mutation bias at the third codon site. An A-ending codon has nucleotide frequency of nucleotide A; ^(3)^ Spike protein gene in reference SARS-CoV-2 genome (NC_045512) and BNT-162b2.

**Table 2:** Usage of CGC and CGG codons in the most highly expressed genes in skeletal muscles. Only those CDSs with the number of codons (N_codon_) greater than 300 were included.

Gene	CGC	CGG	N_codon_	Gene	CGC	CGG	N_codon_
TTN	140	120	35,992	DES	20	13	471
NEB	61	42	8561	ANKRD2	6	9	447
FLNC	47	43	2726	ENO3	7	2	444
MYH2	19	16	1942	ALDOA	9	2	419
MYH1	23	14	1940	PDK4	4	3	412
MYH7	37	38	1936	CKM	9	4	382
MYBPC1	9	5	1196	ACTA1	12	0	378
ATP2A1	11	19	1002	YBX3	11	10	373
PYGM	24	21	843	PDLIM3	3	6	365
UBC	8	0	686	FHL1	4	2	340
KLHL41	3	2	607	GAPDH	2	0	336
PKM	8	9	606	TPM2	10	2	304
BIN1	5	5	594	MYOZ1	1	2	300

**Table 3:** Codon optimization of Asp (D) codons in the two vaccine mRNAs. The column headers are identical to those in Table 1.

AA	Codon	RP	Bkground	S_Ref_	S_BNT-162b2_	S_mRNA-1273_
E	GAA	331	0.2640	34	14	0
E	GAG	426	0.2262	14	34	48

**Table 4:** Codon adaptation index (CAI) and index of translation efficiency (I_TE_) for the coding sequences of the S gene from SARS-CoV-2 reference genome (NC_045512), SARS-CoV reference genome (NC_004718), their close relatives isolated from bats and pangolin, and the two mRNA vaccines (BNT-162b2 and mRNA-1273).

Name	Length	CAI	I_TE_^(1)^
NC_045512_SARS_CoV_2	3819	0.68767	0.5616
MN996532_Bat_RaTG13	3807	0.68657	0.5598
pangolin|EPI_ISL_410721|2019	3795	0.68737	0.5604
MG772933_Bat_SARS-like	3738	0.69885	0.5758
MG772934_Bat_SARS-like	3735	0.69697	0.5697
NC_004718_SARS	3765	0.69593	0.5735
BNT-162b2	3819	0.94925	0.8989
mRNA-1273	3819	0.97939	0.9569

(1) Calculated with DAMBE [20] with codon usage table of “Homo_sapiens_HEG_RibosomalProteins”.

**Table 5:** Site-specific frequencies (columns 2–5) of 11,327 CDSs (including isoforms) in human chromosome 1 (NC_000001.11), and the position weight matrix (columns 6–9) derived from them using intron nucleotide frequencies (0.26398, 0.21777, 0.22622 and 0.29203 for A, C, G, and T, respectively) as background frequencies. Favored nucleotides are highlighted in bold. Start codons are at sites 7–9. The favored translation initiation motif is GCCACC**AUG**GCG.

Site	A	C	G	U	A	C	G	U
1	2437	2642	**4065**	2183	−0.2951	0.0991	**0.6657**	−0.5995
2	2471	**3168**	3070	2618	−0.2751	**0.361**	0.2607	−0.3374
3	2649	**3907**	2947	1824	−0.1747	**0.6634**	0.2017	−0.8587
4	**4997**	1563	3677	1090	**0.7408**	−0.6582	0.521	−1.6013
5	3462	**3493**	2577	1795	0.2114	**0.5019**	0.0082	−0.8818
6	2246	**4740**	3315	1026	−0.4128	**0.9423**	0.3715	−1.6885
7	**11,325**	2	0	0	**1.9212**	−10.1194	−13.4676	−13.4676
8	0	1	0	**11,326**	−13.4676	−10.9842	−13.4676	**1.7756**
9	2	0	**11,325**	0	−10.3672	−13.4676	**2.1438**	−13.4676
10	2933	1723	**4950**	1721	−0.0278	−0.5176	**0.9498**	−0.9425
11	3053	**4218**	2075	1981	0.03	**0.7739**	−0.3044	−0.7395
12	1929	2800	**4072**	2526	−0.6323	0.1828	**0.6682**	−0.389

**Table 6:** Highly expressed genes prefer UAA stop codon. Human chromosome 1 (Chr01) contains 11,327 coding sequences (CDSs, including splicing isoforms), which is divided into two groups based on proportion of nucleotides C and G (P_CG_) in the CDS. Stop codon usage of these CDSs change with P_CG_ and differ from highly expressed ribosomal protein genes (RP, including splicing isoforms).

Group	P_CG_	n	UAA	UAG	UGA
Chr01	≤0.52	5940	0.3828	0.2130	0.4042
	>0.52	5387	0.1916	0.2437	0.5647
RP	≤0.52	347	0.7406	0.1527	0.1066
	>0.52	309	0.4563	0.2201	0.3236

**Table 7:** Contrasts between highly expressed ribosomal protein genes (RP) and all CDSs in human chromosome 1 (Chr01, including isoforms). Shown are the total number of individual stop codons (n) and the percentage of +4A, +4C, +4G, and +4U (P_+4A_, P_+4C_, P_+4G_, and P_+4U_) for CDSs terminating with UAA, UAG and UGA.

	Chr01			RP		
	UAA	UAG	UGA	UAA	UAG	UGA
n	3306	2578	5443	236	104	100
P_+4A_	38.02	28.86	27.10	94.92	34.62	60.00
P_+4C_	25.71	28.94	34.94	13.14	1.92	28.00
P_+4G_	16.64	26.11	22.74	60.17	89.42	60.00
P_+4T_	19.63	16.10	15.21	26.69	8.65	12.00

## Data Availability

Not applicable.

## Supplementary Materials

The following are available online at https://www.mdpi.com/article/10.3390/vaccines9070734/s1, Ribosomal protein-coding genes: RP_Longest_isoform_ubiquitous.fas; Highly expressed genes in skeletal muscle: HEG50_Muscle.fas.
